# Discovery and Validation of Prognostic Biomarker Models to Guide Triage among Adult Dengue Patients at Early Infection

**DOI:** 10.1371/journal.pone.0155993

**Published:** 2016-06-10

**Authors:** Junxiong Pang, Anna Lindblom, Thomas Tolfvenstam, Tun-Linn Thein, Ahmad Nazri Mohamed Naim, Ling Ling, Angelia Chow, Mark I-Cheng Chen, Eng Eong Ooi, Yee Sin Leo, Martin L. Hibberd

**Affiliations:** 1 Infectious Disease, Genome Institute of Singapore, Singapore, Singapore; 2 Saw Swee Hock School of Public Health, National University of Singapore, Singapore, Singapore; 3 Communicable Diseases Center, Institute of Infectious Diseases and Epidemiology, Tan Tock Seng Hospital, Singapore, Singapore; 4 Department of Medicine Solna, Unit of Infectious Diseases, Center for Molecular Medicine, Karolinska Institutet, Karolinska University Hospital, Stockholm, Sweden; 5 Unit for Highly Pathogenic Viruses, Public Health Agency of Sweden, Stockholm, Sweden; 6 Program in Emerging Infectious Diseases, Duke-NUS Graduate Medical School, Singapore, Singapore; 7 DSO National Laboratories, Singapore, Singapore; 8 Department of Medicine, Yong Loo Lin School of Medicine, National University of Singapore, Singapore, Singapore; 9 London School of Hygiene & Tropical Medicine, London, United Kingdom; Northwestern University Feinberg School of Medicine, UNITED STATES

## Abstract

**Background:**

Dengue results in a significant public health burden in endemic regions. The World Health Organization (WHO) recommended the use of warning signs (WS) to stratify patients at risk of severe dengue disease in 2009. However, WS is limited in stratifying adult dengue patients at early infection (Day 1–3 post fever), who require close monitoring in hospitals to prevent severe dengue. The aim of this study is to identify and validate prognostic models, built with differentially expressed biomarkers, that enable the early identification of those with early dengue infection that require close clinical monitoring.

**Methods:**

RNA microarray and protein assays were performed to identify differentially expressed biomarkers of severity among 92 adult dengue patients recruited at early infection from years 2005–2008. This comprised 47 cases who developed WS after first presentation and required hospitalization (WS+Hosp), as well as 45 controls who did not develop WS after first presentation and did not require hospitalization (Non-WS+Non-Hosp). Independent validation was conducted with 80 adult dengue patients recruited from years 2009–2012. Prognostic models were developed based on forward stepwise and backward elimination estimation, using multiple logistic regressions. Prognostic power was estimated by the area under the receiver operating characteristic curve (AUC).

**Results:**

The WS+Hosp group had significantly higher viral load (P<0.001), lower platelet (P<0.001) and lymphocytes counts (P = 0.004) at early infection compared to the Non-WS+Non-Hosp group. From the RNA microarray and protein assays, the top single RNA and protein prognostic models at early infection were *CCL8* RNA (AUC:0.73) and IP-10 protein (AUC:0.74), respectively. The model with *CCL8*, *VPS13C* RNA, uPAR protein, and with *CCL8*, *VPS13C* RNA and platelets were the best biomarker models for stratifying adult dengue patients at early infection, with sensitivity and specificity up to 83% and 84%, respectively. These results were tested in the independent validation group, showing sensitivity and specificity up to 96% and 54.6%, respectively.

**Conclusions:**

At early infection, adult dengue patients who later presented WS and require hospitalization have significantly different pathophysiology compared with patients who consistently presented no WS and / or require no hospitalization. The molecular prognostic models developed and validated here based on these pathophysiology differences, could offer earlier and complementary indicators to the clinical WHO 2009 WS guide, in order to triage adult dengue patients at early infection.

## Introduction

Dengue results in a significant public health burden in the endemic regions, particularly in the World Health Organization (WHO) South-East Asia and Western Pacific Regions, accounting for nearly 75% of the current global dengue disease burden [1–3]. Over decades, there is an increasing trend of young adults requiring hospitalization due to dengue [4–7]. Specific antiviral therapy is not available, making case management entirely supportive and a vaccine, is currently not widely available. The standard-of-care is directed towards constant monitoring of patients with the aim of providing appropriate and timely fluid support, to prevent the development of hypovolemic shock [3,8]. Besides the importance of early dengue diagnosis, an early prognostic tool that predicts dengue severity and guides clinical triage to reduce severe dengue progression and over-hospitalization is also critical [5,9–11]. In 2009, the WHO introduced a revised dengue classification advocating clinicians to look out for clinical presentation of specific WS during triage, as indicators of possible severe dengue progression and to recommend strict monitoring[1].

However, there are a number of challenges encountered when applying the WS-guided dengue classification among adult dengue patients. Firstly, it was reported to be too sensitive and not specific enough in identifying severe illness, resulting in a significant increase in hospitalization, workload of medical personnel and economic burden for resource-limited endemic regions [6,12,13]. Secondly, WS generally occurred only one day prior to the development of severe illness/ requirement of intervention, at 4–7 days post fever onset (p.f.), and this narrow window makes any form of intervention challenging, particularly when appropriate healthcare facilities are not accessible or available near their place of residences [6,8]. An earlier identification of dengue patients who are at high risk of severe disease could also benefit the introduction of therapeutic interventions, when developed [14], offering a longer window for therapy response. Lastly, although the presence of any WS was strongly associated with severe outcome, no single WS can independently predict disease progression, and hence the requirement for close monitoring in hospital in order to fully characterize the WS. Only patients with no WS were highly predictive of non-severe dengue outcomes and could be safely managed as outpatients [15,16]. As such, further refinement of the triage process at the early infection stage for patients who are likely to develop WS later and require hospitalization, would be particularly useful in the primary healthcare setting to reduce dengue burden [12,17].

As the expression of host RNA and proteins are dynamic and sensitive to stimulus from changes in environment, diet, metabolism as well as pathogen infection, the systematic analysis of molecular features has been widely adopted for biomarker model discovery. Once identified and validated, biomarkers of clinical value could be incorporated into numerous types of predictive tools, but preferably into rapid, point-of-care tests. [18,19].

In this study, we aim (1) to identify early molecular features predicting WS and hospitalization requirement, (2) to build biomarker models for close monitoring requirement in hospitals, (3) to evaluate performance of biomarker models to stratify patients at Day 1–3 p.f. who are at higher risk of developing WS later and require hospitalization and (4) to perform an independent validation for the top optimal prognostic models.

## Methods

### Study sites and population studied

**Fig 1:**
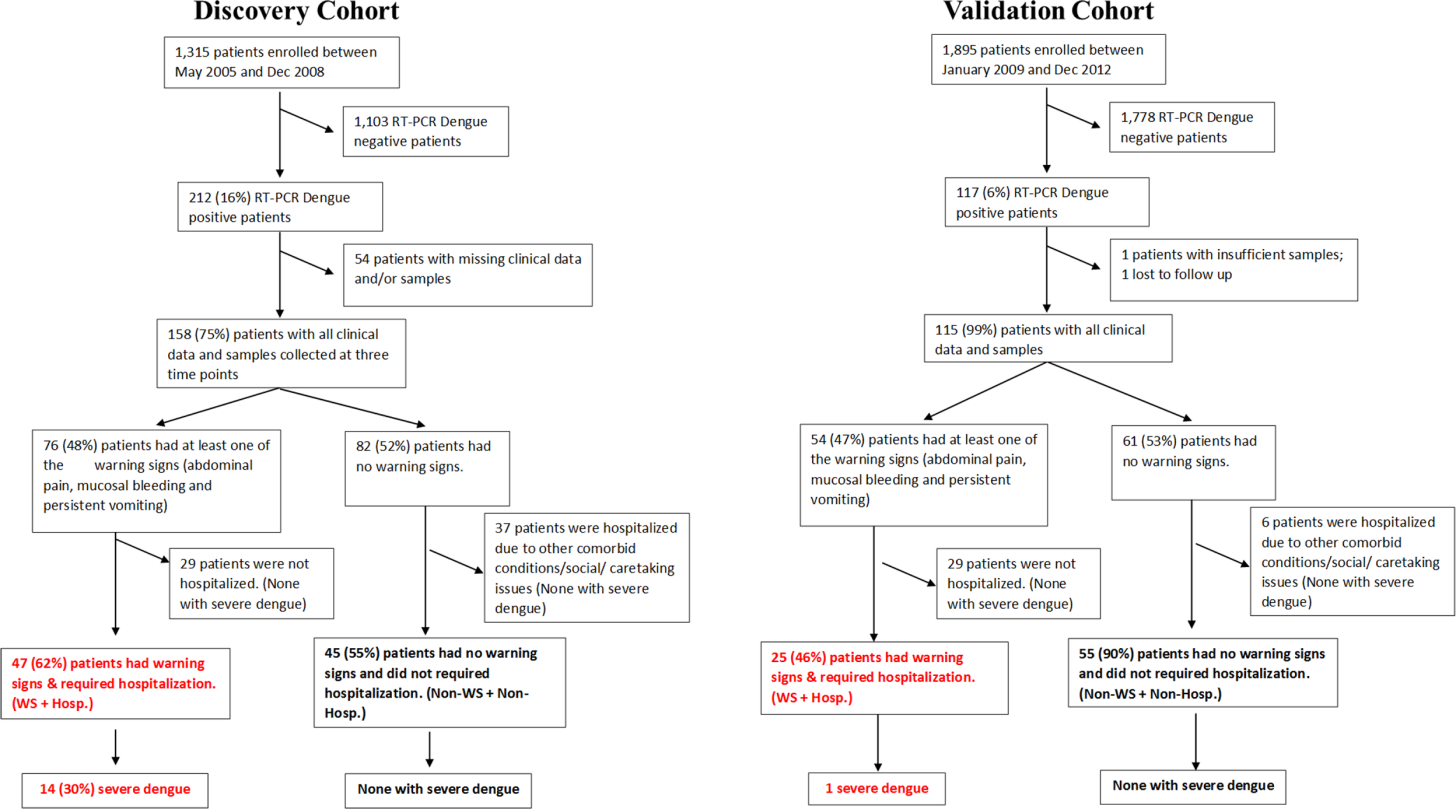
Selection workflow of dengue patients with warning signs that required hospitalizations and patients with no warning signs and no hospitalization required for both discovery and validation cohort.

The participants in the discovery cohort were ≥18 years of age who presented within 72 hrs from acute onset of fever of 38°C or above, with no clinically obvious alternative diagnosis to fever at the participating primary care polyclinics in Singapore, as part of the early dengue infection and control study (EDEN) conducted from years 2005–2008, described in previous publications [20–22]. The participants in an independent validation cohort also had the same inclusion and exclusion criteria but were recruited from years 2009–2012. Participants who had a positive dengue polymerase chain reaction (PCR) test were included in this study. Blood and serum samples from the participants were collected at three time points (at Day 1–3 p.f., Day 4–7 p.f. and three to four weeks p.f.). These study cohorts are outlined in Fig 1.

### Dengue classification and warning signs criteria

The 2009 WHO dengue severity classification based on clinical signs and symptoms were applied in this study. This divides patients into “probably dengue”, “dengue with warning signs (WS)” and “severe dengue”. The WS stated in the guidelines are; abdominal pain or tenderness, persistent vomiting, clinical fluid accumulation, mucosal bleed, lethargy, restlessness, persistent vomiting, liver enlargement >2cm and increase in HCT concurrent with rapid decrease in platelet account. Severe dengue was defined by severe plasma leakage, severe bleeding and/or severe organ impairment [1]. In the EDEN study, only three WS were recorded during the study period from year 2005–2008. They were abdominal pain, mucosal bleeding and persistent vomiting. Persistent vomiting was defined as vomiting at two study clinic visits and/or one study clinic visit and during hospitalization. Severe plasma leakage was defined as either a pulse pressure difference of less than 20 mmHg, a systolic pressure of less than90 mmHg in need of intervention, pleural effusion or ascites. Pleural effusion and ascites was diagnosed with chest x-ray/ultrasound. Severe bleeding was defined as internal bleeding requiring transfusion. Severe organ impairment was considered as liver AST or ALT >1000 and CNS: impaired consciousness.

### Hospital admission criteria

The decision to hospitalize a patient was left to the discretion of the treating physician. However, national guidelines on dengue management are available and are adopted by the healthcare institutions in Singapore. Hospitalization criteria in these guidelines include: significant bleeding, fall in blood pressure, dehydration and postural hypotension, rise in hematocrit of 20% or greater compared to the baseline, platelet count <80,000 cells/mm3, severe vomiting or diarrhea, severe abdominal pain, and elderly patients with co-morbidities who are unwell.

### Clinical data and sample management

A standardized case report form (CRF) was used for collecting clinical data. Additionally, venous blood for hematological and molecular analyses was collected. Clinical data and samples were obtained at time of inclusion (within 72 hrs post fever onset), at time of defervescence (4–7 days post fever onset) and finally at time of convalescence (3–4 weeks post fever onset). The clinical data for hospitalized patients were obtained from the electronic medical records.

### Hematology and serology

A full blood count was performed on anticoagulated whole blood collected at all three time points. A bench-top, FDA-approved hematocytometer was used for this application (iPoch-100, Sysmex, Japan). Calibration by internal and external QC controls was also performed on a regular basis. IgM and IgG antibodies against dengue virus were detected using commercially available ELISAs (PanBio, Brisbane, Australia) according to manufacturer’s instructions.

### Viral detection and quantification

RNA was extracted using QIAamp Viral RNA mini kit (Qiagen, Hilden, Germany) according to the manufacturer’s protocol. Dengue virus RNA detection was carried out by PCR using a set of generic pan-dengue primers that targeted the 3’ non-coding region of dengue viruses as previously described [23]. Results were analyzed with the Light Cycler software version 3.5. Reactions with high crossover threshold (Ct) value or ambiguous melting curve results were further analyzed by electrophoresis on a 2% agarose gel, to confirm the presence of the correctly sized amplicon. Quantification of viremia was performed by a Taqman based PCR using earlier published primers and probes detecting DENV 1–4 [24]. Standard ABI conditions were used, incorporating primers at 900nM and probes at 50nM.

### Microarray

Total RNA (500ng) was amplified in a single-round of IVT amplification that allowed incorporation of Biotin-labeled nucleotides using the Illumina® TotalPrep™ RNA Amplification Kit (Ambion, Inc., Austin, TX) according to the manufacturer’s instructions. cRNA (850ng) of each sample was hybridized to an Illumina HumanRef-8 V3.0 BeadChip following the manufacturer’s instructions (Illumina, Inc., San Diego, CA). This was followed by washing, blocking, and streptavadin-Cy3 staining steps and finally by scanning with a high resolution Illumina Bead Array Reader confocal scanner, all carried out following manufacturer’s instructions (Illumina, Inc., San Diego, CA).

### Microarray normalization and gene selection

The detection *p*-value was calculated by Beadstudio software (Illumina). Standard normalization procedures (GenespringGX software; version10.0; Silicon Genetics) for one colour array data were used. In brief, array (mean) normalization accounted for chip variability was performed by dividing all of the measurements on each chip by a 75^th^ percentile value. After normalization, the data was filtered according to flags present there at least 75% of the samples in any 1 of the 2 conditions had flags present leaving 6844 genes for further analyzes. Significance Analysis of Microarray (SAM) was used to detect transcripts that were relatively more or less abundant in one group of samples. SAM also corrected significance values for multiple testing using a false discovery rate threshold of 5%. False discovery rate of less than 5 percent and fold difference of at least 1.5 fold were used to identify the significant genes. Pathway analysis was done using Ingenuity Pathway Analysis software (version 7.5; Ingenuity Systems).

### Measurement of RNA expression using Fluidigm technology

In order to develop a potential point-of-care device, a simple PCR based technology should be applied instead of using a microarray based technology, to reduce cost and processing time. Fluidigm platform was used. The required amount of RNA is 500ng/10ul per reaction. The protocols were according to the manufacturer’s recommended instructions. Briefly, cDNA is synthesized through reverse transcription using MultiScribe reverse transcriptase with the following program: 25°C for 10 min, 37°C for 120 min, 85°C for 5 min and 4°C for 30min. This is followed by pooling of Taqman assays and pre-amplification reaction with the following program: 95 for 10min, and 14°C cycles of 95°C for 15sec and 60°C for 4 min. Lastly, this is followed on with final amplification using the BioMark and the 48.48 dynamic array as instructed in the manufacturer’s instructions. The signal of the gene expression was normalised by 18S rRNA expression. The expression level and quality control checks were determined using the BioMark Real-Time PCR Analysis Software.

### Protein measurements

Serum samples collected were assayed for 22 cytokines and chemokines (S2 Table) related to inflammation and immune using a luminex bead array approach (Bioplex) (BioRad Carlsbad, CA) according to the manufacturer’s protocol. Quantitative sandwich enzyme-linked immunosorbent assays (ELISA) were used to measure fibrinogen (Immunology Consultants Laboratory Inc, Newberg, OR), urokinase plasminogen activator receptor (uPAR) as well as IP-10 (R&D Systems, Minneapolis, MN). The assay from R&D Systems was needed as a significant number of serum samples from dengue patients had concentration of IP-10 above the detection range of the Bioplex system. All assays were carried out according to the manufacturers’ instructions.

### Statistical analyses

For descriptive analysis, Pearson’s chi-square and Fisher’s exact tests were used to compare categorical variables. Mann-Whitney U test and Student T-test were used to compare continuous variables with non-normal distribution (age and viral load) and normal distribution, respectively. Univariate and multivariate logistic regression were used to calculate crude and adjusted odds ratios (COR; AOR), respectively with 95% confidence intervals (CI) reported. P-value of <0.05 was considered as statistically significant. All statistical analyses were performed using Stata 10.0 (Stata Corp., College Station, TX, 2005).

### Biomarkers selection and prognostic model development

RNA and proteins biomarkers that were significantly different (P<0.05) between the two groups at Day 1–3 p.f. were selected and ranked accordingly from the most significance (P<0.01) to the least significance (P = 0.05) for the model development. Model development was based on forward stepwise and backward elimination estimation using multiple logistic regressions. Prognostic performance was based on the area under the receiver operating characteristic curve (AUC), Hosmer-Lemeshow Goodness-Of-Fit (GOF) test, and likelihood ratio. Models were also compared with the inclusion of three laboratory variables (viral load, platelets, and lymphocytes), as well as the three warning signs (abdominal pain, mucosal bleeding and persistent vomiting) that are recorded in the EDEN study from 2008–2009.

### Ethical approval

The study protocol was approved by the National Healthcare Group Domain Specific Review Board (DSRB B/05/013), as well as the Institutional Review Boards of the National University of Singapore and DSO National Laboratories. Enrolment of study participants was conditional on appropriate written informed consent administered by designated qualified study research nurse.

## Results

Between May 2005 and December 2008, a total of 1,315 suspected dengue patients were enrolled. Among the 212 (16%) patients who had RT-PCR confirmed dengue infection, 54 were excluded due to missing data/samples. Of the remaining 158 patients, 76 patients had at least one of the three WS and 82 patients had no WS (Fig 1). There were 47 patients who later developed WS and were subsequently hospitalized (WS+Hosp. Group; Fig 1). These patients were representative of those that should be prioritized for strict monitoring and interventions at early infection according to the 2009 WHO dengue severity classification [1]. Furthermore, 45 patients who did not develop WS and were not hospitalized (Non-WS+Non-Hosp. Group; Fig 1) represented a group of mild dengue patients that were safely managed as outpatients. Twenty-nine patients with warning signs were not hospitalized and 37 patients without warning signs were hospitalized (Fig 1).

All these patients were followed longitudinally at our study clinics for the scheduled three visits. Of the 47 patients in the WS+Hosp. Group, 14 (30%) progressed to severe disease as defined by the 2009 WHO dengue severity classification [1].None of the patients in the Non-WS+Non-Hosp. Group and the not hospitalized patients with WS developed severe dengue disease (Fig 1). Five out of 37 (14%) patients in the group of hospitalized patients without WS developed severe dengue. No deaths were reported in any of the groups. Participants who later developed WS but were not hospitalized were excluded from our discovery cohort as it was not possible to undertake detailed severity assessments without serial daily measurements. Moreover, participants who did not develop WS but were hospitalized, were excluded as they were likely admitted due to non-dengue concerns (such as deterioration of other co-morbid conditions) or on non-clinical grounds (such as lack of adequate care at home).

### Clinical and laboratory characteristics of discovery cohort

Among the WS+Hosp. group, 44 (94%), 10 (21%) and 5 (11%) reported mucosal bleeding, persistent vomiting and abdominal pain, respectively. Only six (14%) patients had signs of mucosal bleeding at Day 1–3 post fever onset (p.f.), while 14 (32%) had signs at Day 4–7 p.f., and 27 (61%) had signs at time of hospitalization. Moreover, 32 (73%) showed signs of bleeding during hospitalization. Gum bleeding was the most common (n = 18) followed by skin (n = 12), menstrual bleeding (n = 8), nose bleeding (n = 5), hematuria (n = 3) and per rectal bleeding (n = 2). Six patients reported persistent vomiting at both Day 1–3 p.f. and Day 4–7 p.f., while nine reported it at Day 4–7 p.f. and during hospitalization. Two patients had abdominal pain at Day 1–3 p.f. while three patients had abdominal pain at Day 4–7 p.f. The patients in the WS+Hosp. group were admitted at a median of 4 days (range 1–7) p.f. and hospitalized for a median of 3 days (range 1–7) (Table 1). Among patients with severe dengue, 6 had severe plasma leakage, 5 had severe bleeding (2 hematuria, 3 rectal bleeding) and 3 had severe organ involvement (transaminase >1000 U/L). During hospitalization, 44 out of 47 (94%) patients received intravenous fluid replacement.

**Table 1:** Demographic descriptions of dengue RT-PCR positive patients classified according to designated clinical outcomes.

	EDEN 2005–2008 (Discovery Cohort)					EDEN 2009–2012 (Validation Cohort)				
	Non-WS + Non-Hosp.		WS + Hosp.			Non-WS + Non-Hosp.		WS + Hosp.		
	(N = 45)	%	(N = 47)	%	p-value	(N = 55)	%	(N = 25)	%	p-value
**Age**										
Median (Range)	41 (21–63)		37 (19–77)		0.229^#^	33 (25.5–42.5)		41 (25–52)		0.211^#^
**Gender**										
Female	22	48.9	23	48.9	0.996	7	12.7	10	40	**0.006**
**Ethnicity**										
Chinese	32	71.1	39	83.0		31	56.4	16	64.0	
Malay	1	2.2	4	8.5		5	9.1	6	24.0	
Indian	7	15.6	3	6.4		8	14.5	1	4.0	
Others	5	11.1	1	2.1	0.082	11	20.0	2	8.0	0.122
**Pre-Existing Comorbid**										
Yes	7	15.6	6	12.8	0.701	3	5.5	4	16	0.196^
**Serotype**										
1	16	35.6	25	53.2		3	5.5	3	12	
2	13	28.9	14	29.8		35	63.6	19	76	
3	16	35.6	8	17.0		0	0	2	8	
4	0	0	0	0	0.098	6	10.9	1	4	0.011
Unknown	0	0	0	0		11	20	0	0	
**IgG Status at Presentation**										
Positive	20	44.4	25	53.2	0.401	20	36.4	17	68	**0.009**
**Hospitalization**										
Median days p.f. on admission (Range)	n.a.		4 (1–7)			n.a.		5 (1–8)		
Length of stay (Range)	n.a.		3 (1–7)			n.a.		4 (2–9)		
**Severe Disease**										
Yes	0	0	14	30.0	**<0.001**^	0	0	1	4	0.312^

^#^ Mann-Whitney Test

^ Fisher’s Exact Test

p.f.—post fever onset

n.a.–not applicable

Statistically significant p-values are in bold

**Fig 2:**
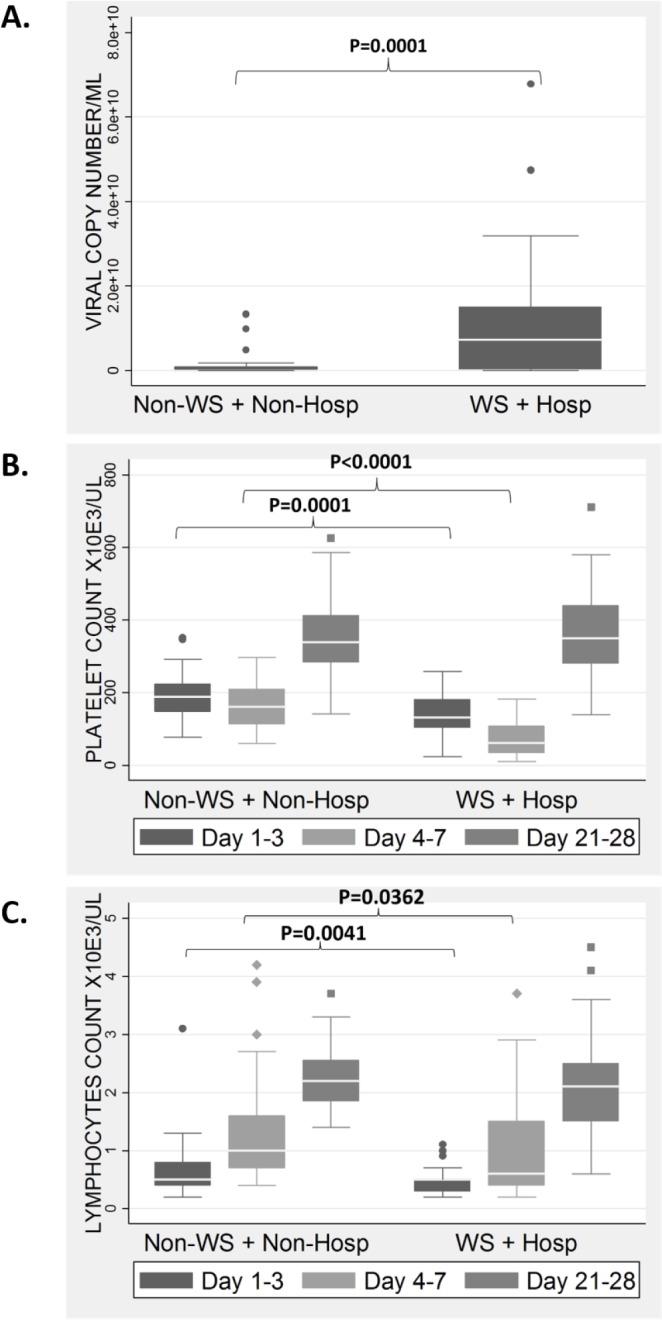
**Laboratory characteristics (A-Viral copy number at Day 1–3; B-Platelet count; C-Lymphocytes count) of hospitalized dengue patients with warning signs (WS + Hosp. Group) compared to non-hospitalized patients with no warning signs (Non-WS + Non-Hosp. Group).** P-value (P) is shown only for statistically significant comparisons on Day 1–3 and Day 4–7 between the two groups.

There were significant differences (P<0.05) in viral, platelet and lymphocyte levels between the WS+Hosp. and Non-WS+Non-Hosp. groups at Day 1–3 p.f. (Fig 2). Within the WS+Hosp. group, there was no significant differences in viral, platelet and lymphocyte levels at Day 1–3 p.f. between the 14 patients who progressed to severe dengue and the remaining 33 patients with no progression to severe dengue (S1 Fig). However, severe dengue patients showed the same significantly higher viral, lower platelet and lymphocyte levels compared to the Non-WS+Non-Hosp; as the WS+Hosp. group did (S2 Fig). This illustrates the similar pathophysiological characteristics at Day 1–3 between patients with WS that develop severe dengue and those patients with WS who do not. However, there are clear differences in pathophysiological characteristics between the Non-WS+Non-Hosp. group compared to both the WS+Hosp. group and the WS+Hosp. patients who progress to severe dengue, suggesting that pathophysiological characteristics can be used to distinguish these groups (independently of WS).

### Differential biomarkers between WS+Hosp. group and Non-WS+Non-Hosp. group at early infection

A total of 23 RNA biomarkers were differentially expressed at Day 1–3 p.f. with ≥1.5 fold difference (P<0.05; S1 Table) between the WS+Hosp. group and the Non-WS+Non-Hosp. group. Eight of these 23 genes are related to innate immune activation, namely *CCL2*, *CCL3*, *CCL8*, *CD69*, *NFIL3*, *RIN2*, *CYP27A1* and *CDKN1C*. Among the immune-related biomarkers, only IL-8, *CCL2*, *CCL3* and *CCL8* were differentially expressed between Non-WS+Non-Hosp. group and severe dengue group (P<0.05; S1 Table).

Out of the 22 protein biomarkers analyzed, the WS+Hosp. group had significantly higher level of four proteins at Day 1–3 p.f. than the Non-WS+Non-Hosp. group, namely interferon gamma-induced protein (IP)-10 (P = 0.0001), interleukin (IL)-1ra (P = 0.0094), fibrinogen (FGA) (P = 0.0423) and urokinase-type plasminogen activator receptor (uPAR) (P = 0.0047) (S2 Table). The WS+Hosp. group had significantly lower level (P = 0.0207) of RANTES than the Non-WS+Non-Hosp. group at Day 1–3. However, only CCL4, IP-10 and uPAR were differentially expressed between Non-WS+Non-Hosp. group and severe dengue group (P<0.05; S3 Table).

#### Prognostic performance of RNA and protein biomarker models at early infection

**Fig 3:**
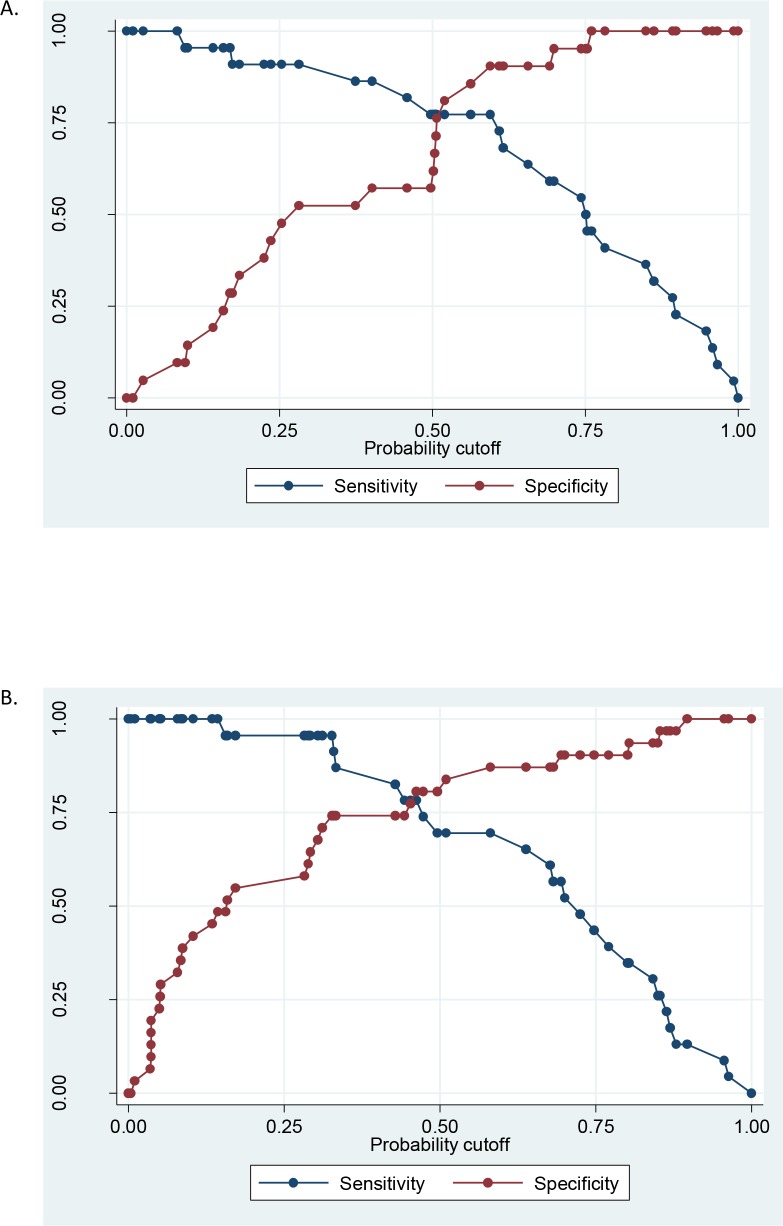
**Sensitivity and specificity plots of Model 13 (A) and Model 14 (B) with the varying probability cut-offs using the discovery cohort.** The y-axis shows the true positive rate (sensitivity in blue) and the true negative rate (specificity in red) of the model’s capability at different probability cutoff on the x-axis. The probability cutoff range (x-axis) allows the investigators to choose how sensitive and specific they want the model to be at different setting depending on the aim of the models.

The IP-10 protein (Model 1; AUC = 0.74) and *CCL8* RNA (Model 3; AUC = 0.73) biomarkers were the top single biomarker models for protein and RNA, respectively (Table 2). Among the multiple biomarker models developed (and shown in Table 2), Model 13 (*CCL8*, *VPS13C* RNA, and uPAR protein) and Model 14 (*CCL8*, *VPS13C* RNA, and Platelets Level) had the greatest AUC of about 0.90 and 0.88, respectively, and both were more parsimonious than Model 12 (*HIST14HE*, *VPS13C* RNAs, and IL-1RA, uPAR proteins). With the probability cutoff at 0.5, Model 13 had a sensitivity of 82.9%, specificity of 80.0%, positive predictive value (PPV) of 80.6% and a negative predictive value (NPV) of 82.4%; while Model 14 had sensitivity of 80.9% sensitivity, 84.4% specificity, PPV of 80.6% and a NPV of 82.4%. The sensitivity and specificity may be optimized by varying the probability cut-off, as shown in Fig 3.

**Table 2:** Early prognostic models of warning signs and hospitalization from the discovery cohort.

Model	Variables (*RNA*/Proteins/Lab/Warning Signs)	AUC	Sen (%)	Spe (%)	PPV (%)	NPV (%)	GOF test (p-value)
	**Models without Laboratory Features**						
**1**	**IP-10**	**0.7353**	**54.35**	**77.78**	**71.43**	**62.50**	**0.57**
2	IL-1ra	0.7348	55.32	84.44	78.79	64.41	0.29
**3**	***CCL8***	**0.7277**	**72.34**	**66.67**	**69.39**	**69.77**	**0.14**
4	*HIST1H4E*	0.7229	76.60	55.56	64.29	69.44	0.06
5	*PKD2L1*	0.7047	72.34	53.33	61.82	64.86	0.68
6	*CCL3*	0.6979	63.83	64.44	65.22	63.04	0.57
7	uPAR	0.6955	60.00	74.29	70.00	65.00	0.77
8	*VPS13C*	0.6950	61.70	53.33	58.00	57.14	0.05
9	*RGL1*	0.6946	68.09	53.33	60.38	61.54	0.70
10	*NCOA7*	0.6927	70.21	60.00	64.71	65.85	0.13
11	IP-10, *CCL8*	0.7942	71.74	73.33	73.33	71.74	0.48
12	*HIST14HE*, *VPS13C*, IL-1RAuPAR*	0.9045	82.86	77.14	78.38	81.82	0.62
**13**	***CCL8*, *VPS13C*, uPAR**^^§^	**0.8988**	**82.86**	**80.00**	**80.56**	**82.35**	**0.24**
	**Models with Laboratory Features**						
**14**	***CCL8*, *VPS13C*, Platelets Level**^*	**0.8757**	**80.85**	**84.44**	**84.44**	**80.85**	**0.32**
15	Platelets Level	0.7390	72.34	68.89	70.83	70.45	0.84
16	Viral Ct Level	0.7058	73.91	56.82	64.15	67.57	0.56
17	Lymphocytes Level	0.6792	76.60	48.89	61.02	66.67	0.01#
18	Platelets and Viral Ct Level	0.8370	73.91	79.55	79.07	74.47	0.20
19	Platelets, Viral Ct Level, IP10	0.8520	77.78	81.82	81.40	78.26	0.08
20	Platelets Level, IP10	0.8097	76.09	77.78	76.09	77.78	0.33
21	Platelets, Viral Ct Level, *CCL8*	0.8696	84.78	79.55	81.25	83.33	0.02#
	**Models with Warning Signs**						
22	Abdominal Pain	0.5102	100.0	0	51.09	-	N.A
23	Persistent Vomiting	0.5615	23.40	88.89	68.75	52.63	N.A
24	Mucosal Bleeding	0.5000	0	100	-	52.33	N.A
	**Internal Validation using Fluidigm for *CCL8*, *VPS13C***						
13	***CCL8*, *VPS13C*, uPAR**	**0.8420**	**77.27**	**61.90**	**68.00**	**72.22**	**0.34**
14	***CCL8*, *VPS13C*, PLT**	**0.8682**	**69.57**	**83.87**	**76.19**	**78.79**	**0.74**
11	IP10, *CCL8*	0.7451	41.18	88.89	70	70.59	0.83

Sen- Sensitivity; Spe- Specificity; PPV- Positive predictive value; NPV- Negative predictive value; N.A.- Not applicable. Sen, Spe, PPV and NPV are based on probability cutoff of 0.5.

^ Forward Stepwise Estimation from top 10 single RNA and protein molecules based on AUC

* Backward Elimination Estimation from top 10 single RNA and protein molecules based on AUC

^§^ Likelihood-Ratio test shows model 13 and 14 provide the same fit as model 12 (p-value>0.05)

^#^Model has significant lack of fit for the data

GOF- Goodness-of-fit test showed significant “lack-of-fit” when p<0.05.

Models which comprised of a single laboratory parameter (platelets, viral, lymphocytes) or warning sign (abdominal pain, persistent vomiting and mucosal bleeding) were not as effective as Model 13 and 14 (Table 2).Even though the multiple biomarker marker Model 21(platelets, viral Ct level, and *CCL8* RNA) had AUC of0.87, it does not fit the dataset well (Goodness-of-fit test P<0.05). In order to evaluate the microarray results for*CCL8*, *VPS13C*RNAs, real-time polymerase chain reaction technology (Fluidigm) was selected to validate the *CCL8*, *VPS13C* RNA levels. Using Fluidigm as an internal validation with the same biological samples, Model 13 and 14 had similar AUC of 0.84 and 0.87, respectively (Table 2). This clearly illustrates the potential of using PCR as a suitable platform for these RNA biomarkers.

### Independent validation of the top two prognostic models

**Fig 4:**
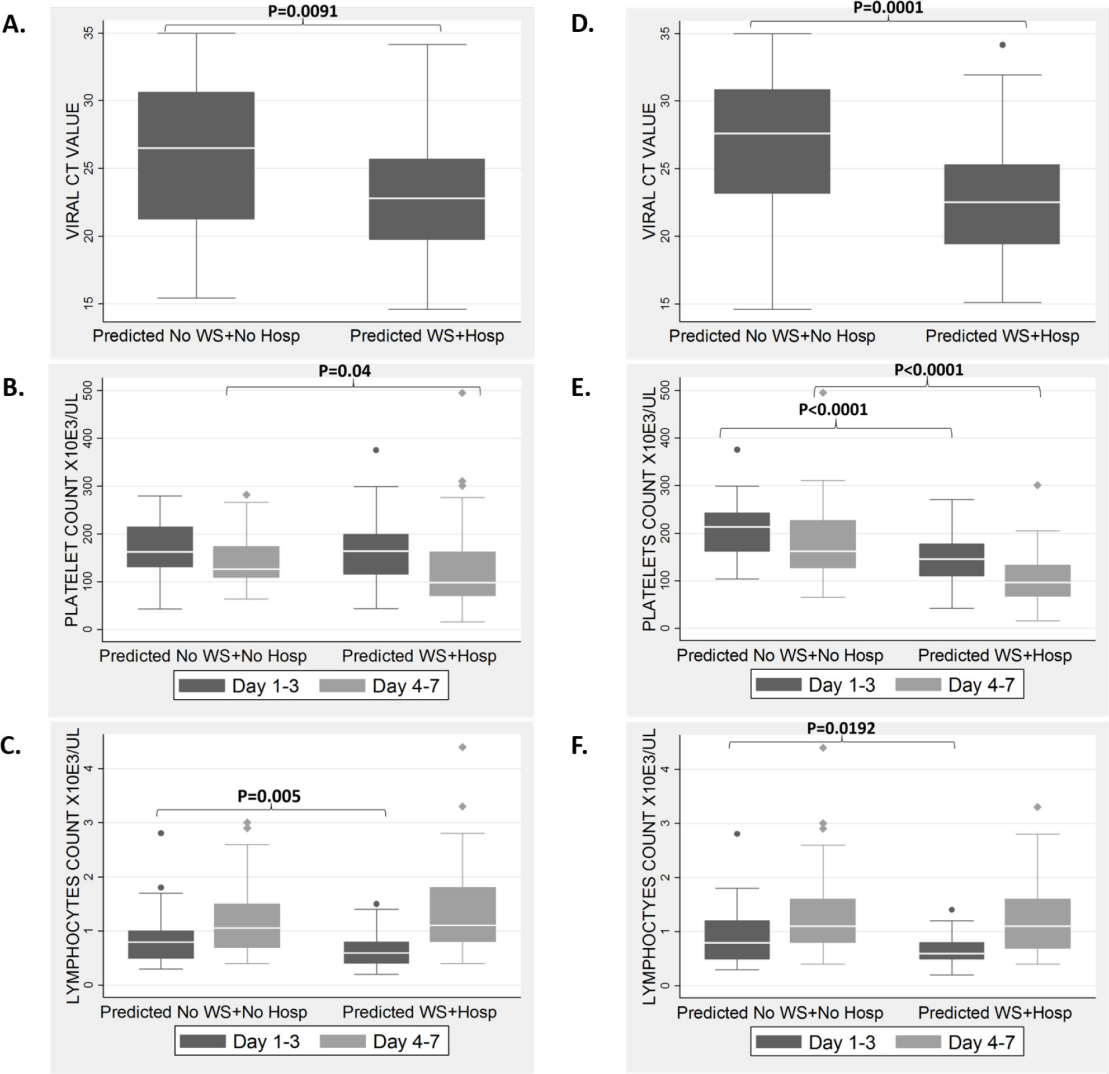
**Laboratory characteristics of patients in the validation cohort who are predicted at Day 1–3 p.f. to develop warning signs and require hospitalization at post Day 1–3 p.f. using Model 13 (A, B and C) and Model 14 (D, E and F).** Viral Ct level at Day 1–3 (A, D), platelet (B, E) and lymphocyte (C, F) levels of the patients who were predicted into either the “Non-WS + Non-Hosp” group or “WS + Hosp” group. WS- Warning Signs; Hosp-Hospitalisation. P-value (P) is shown only for statistically significant comparisons on Day 1–3 and Day 4–7 between the two predicted groups.

Between January 2009 and December 2012, there were 1,895 suspected dengue patients enrolled. Among which, 117 (6%) patients were dengue RT-PCR positive. Using the same inclusion and exclusion criteria as the discovery cohort (described in Fig 1), 25 dengue patients who presented at Days 1–3 p.f. and had WS with hospitalization were classified as the WS+Hosp. group, and 55 dengue patients who presented at Days 1–3 p.f. but did not have WS and had no hospitalization requirement were classified as the Non-WS+Non-Hosp. group. Patient characteristics are outlined in Table 1. Using this independent cohort for validation, model 13 achieved 64% sensitivity, 76% specificity, PPV of 55% and NPV of 82% at Days 1–3 p.f. (Table 3), while model 14 achieved 60% sensitivity, 78% specificity, a PPV of 56% and a NPV of 81% at Days 1–3 p.f. (Table 3). In addition, various probability cutoffs were also assessed. With a probability cutoff of 0.2, the validation resulted in much higher sensitivity of 96% and a modest 55% specificity (Table 3).There was only one patient with severe dengue in this validation cohort. Both models13 & 14 were able to correctly classify this severe dengue patient into the WS+Hosp. group at Days 1–3 p.f. (Well before the patient actually developed WS). We next assessed if the model predictions identified patients that showed the characteristic pathophysiology differences observed in our discovery process. Viral Ct value, platelets and lymphocyte levels were significantly different (P<0.01) between the predicted WS + Hosp. group and the predicted Non-WS+Non-Hosp group in this validation cohort at Day 1–3 p.f. except for platelets level between the two groups predicted by Model 13 (Fig 4). Platelet level between the two groups predicted by Model 13 and 14 were significantly different at Day 4–7 p.f. (Fig 4B and 4E).

**Table 3:** Early prognostic performance of the top selected models with an independent validation cohort across a range of probability cutoff.

Model	Variables	Probability Cutoff	Sensitivity	Specificity	PPV	NPV
1	IP-10	0.2	100.0	0	31.3	-
		0.5	80.0	50.9	42.6	84.9
		0.8	32.0	96.4	80.0	75.7
3	*CCL8*	0.2	96.0	16.4	34.3	90.0
		0.5	48.0	67.3	40.0	74.0
		0.8	8.0	98.2	66.7	70.1
13	*CCL8*, *VPS13C*, uPAR	0.2	96.0	49.1	46.2	96.4
		**0.5**	**64.0**	**76.4**	**55.2**	**82.4**
		0.8	40.0	90.9	66.7	76.9
14	*CCL8*, *VPS13C*, Platelets	0.2	96.0	54.6	49.0	96.8
		**0.5**	**60.0**	**78.2**	**55.6**	**81.1**
		0.8	32.0	94.6	72.7	75.4

## Discussion

The diverse clinical spectrum of dengue disease presentations is still a challenge for health care workers in dengue endemic regions, especially to identify patients early that will later require clinical interventions to prevent progression to severe illness and particularly in adults. While warning signs of the WHO 2009 dengue classification were found to be associated with severe illness, they typically occurred only one day prior to the development of severe illness [6,8,15,16], which would be a challenging window for any effective intervention. In this study, we aimed to identify and validate biomarker models, comprised of distinct molecular features at early dengue infection (Days 1–3 p.f.), that were associated with adult dengue patients who would develop WS and require hospitalization, typically 3 to 5 days later.

The WHO 2009 WS guidelines and clinical judgment were applied to identify patients who did and who did not require clinical observation, dividing the patients into two groups. Those who later developed WS of abdominal pain, bleeding and/or persistent vomiting and were hospitalized (WS+Hosp. group) and those who did not develop WS and did not require hospitalization (Non-WS+Non-Hosp. group). We found that the WS+Hosp. group had significantly higher viremia, but lower platelet and lymphocyte levels compared with Non-WS+Non-Hosp. group at early infection (Days 1–3 p.f.). The Non-WS+Non-Hosp group has to our knowledge not been previously reported in the literature and these novel observations clearly illustrated viral (AUC = 0.71), platelet (AUC = 0.74) and lymphocyte (AUC = 0.68) levels as potential biomarkers to triage patients into the two groups at early infection. Hospitalization typically occurred at Day 4–7, and the platelet and lymphocyte levels were observed to be lower in the WS+Hosp. group than Non-WS+Non-Hosp. group. This suggests that hospitalization due to low platelet and lymphocyte levels was common in our cohort. The lack of statistical differences in viral, platelet or lymphocyte levels between the severe dengue group and the remaining patients from the WS+Hosp. group (S1 Fig) highlights the difficulties in recognizing these severe dengue patients at early infection, as they are likely to be indistinguishable from other hospitalized patients with WS, even on Day 4–7 p.f. This reflects the similar observations between children with severe illness and hospitalized non-severe illness published previously [25]. However, the severe dengue patients were significantly different in pathophysiology from the Non-WS+Non-Hosp. group at early infection (S2 Fig). High viremia has previously been associated with severe dengue outcomes [21,22,26]. In our findings, we showed that early viremia was significantly associated with the later development of WS and the requirement for close hospital monitoring, as was platelet and lymphocyte levels; suggesting an important role of these parameters in the development of dengue disease severity.

In this study, we focused our discovery work on a fully described group of patients with WS and excluded those that were not hospitalized, in order to avoid patients who may have been misclassified. However, it is possible that these excluded patients followed a milder disease. These patients were all followed longitudinally at our study clinics for the scheduled three visits. None of the 29 patients with WS who did not require hospitalization had severe disease as assessed at their third visit compared to 30% of those in the WS+Hosp. group (Fig 1). In addition, the median platelet levels of these 29 patients at the first and second study clinic visit were higher (189 and 139, respectively) as compared to the WS+Hosp. group (132 and 62 respectively) and the hospitalized group of patients without WS (median platelet levels 136 and 77 respectively). This suggests that hospitalization was typically due to low platelet levels by the time of second visit, rather than due to WS in our cohort. To compensate for this potential bias, hospitalized patients without WS were also excluded from our discovery analysis.

The biomarkers associated with the WS+Hosp. group were involved in innate immunity (CCL2, CCL3, CCL8, CD69, RANTES, IL1RA, IP-10) and coagulation (uPAR, FGA) pathways that were previously associated with dengue severity [27–32] andmay be informative of the strength of the innate response during early infection, which may be related to the progression of disease severity after Day 1–3. *CCL8 (MCP-2)*, the top RNA biomarker, is a chemokine that had been previously associated as a biomarker for tuberculosis diagnosis [33] and outcome of hepatitis C virus infection [34]. IP-10 (CXCL-10), the top protein biomarker is a pro-inflammatory chemokine [35], which has been highly associated as a biomarker to predict severity of several inflammatory diseases including infectious diseases, immune dysfunction and tumor development [36]. In our best prognostic analysis, Models 13 & 14, we also utilized uPAR protein and *VPS13C* RNA. Soluble uPAR is a versatile signaling proteinase receptor [37] that had been suggested as a biomarker to predict survival of HIV-1 infection [38] and to discriminate primary focal segmental glomerulosclerosis [39], which may also be related to the protective PLCE1 loci associated with DSS [40]. Furthermore, *VPS13C* RNA encodes for a vascular protein associated with the pathophysiology of type-2 diabetes [41], which may further support the association of diabetes with dengue severity [42]. While these biomarkers showed high biological relevance to dengue pathophysiology, they may not fully explain the development of severe disease, as this may also be influenced by other, as yet undefined, mechanisms.

Many of the WS stated by the WHO 2009 classification are typically seen on Day 4–7 p.f. in the clinical course of disease [6,15,16]. Mucosal bleeding was a common WS in our cohort and in others [6,15], and a majority of the patients showed this WS, mainly during admission into hospital at Day 4–7 p.f. and during hospitalization, which was also observed in other studies [6,43]. Similar to other studies [6,15], our study also showed that some WS, namely abdominal pain (AUC = 0.51), persistent vomiting (AUC = 0.56) and mucosal bleeding (AUC = 0.50), had less optimal prognostic performance in this cohort, reemphasizing the importance to assess the molecular biomarker as a potential prognostic tool.

Ideally, in primary healthcare facilities, the clinician should have a reliable test that can diagnose and predict at Day 1–3 p.f., if a patient had dengue and may progress to severe disease which requires prompt close monitoring and hospitalization. Our findings showed that by combining RNA and protein biomarkers, the best model (*CCL8*, *VPS13C* RNAs, and uPAR protein) gave 82.9% sensitivity, 80.0% specificity in the discovery cohort. Furthermore, by adding platelet counts to the biomarkers, a model (*CCL8*, *VPS13C* RNAs, and Platelets) that gave 81% sensitivity and 84% specificity was established. When validated in an independent cohort, the top two models achieved modest sensitivity and specificity of about 64% and76%, respectively for Model 13 (*CCL8*, *VPS13C* RNAs and uPAR), and 60% and 78%, respectively for Model 14 (*CCL8*, *VPS13C* RNAs and Platelets). However, with the importance of sensitivity in triage, the models may achieve sensitivity and specificity up to 96% and 54.6%, respectively, with a different probability cutoff. Moreover, these models may be tested simultaneously with the dengue virus PCR assay as diagnosis, to additionally guide prompt clinical triage. Furthermore, we showed that by using our models 13 and 14, we were able to accurately predict the expected significant differences in pathophysiology between the WS+Hosp group and the Non-WS+Non-Hosp. group. In addition, both models 13 & 14 are able to identify patients who are likely to present with thrombocytopenia (predicted WS+Hosp. group) or not (predicted Non-WS+Non-Hosp. group) at Day 4–7 p.f. when they first presented to the clinicians at Day 1–3 p.f. with no thrombocytopenia.

The generalizability of these optimal models may be limited until further validation is performed in a larger cohort of adult dengue patients. While the observed different predominant serotype in the discovery and validation cohorts demonstrates some generalizability in the validation group, across both serotypes 1 and 2 viruses, nevertheless, further more diverse studies are required. It may be that single biomarker prediction models such as IP-10 protein and *CCL8* RNA may be more robust when tested in larger number of patients, even though they may not be the most optimal in this study (Table 2). The small proportion of severe dengue patients in our study reflects both the early stage of recruitment and the distinct nature of adult disease [6]. Therefore, it was statistically challenging to develop optimal models in stratifying patients of high risk of severe dengue. Nevertheless, our data highlights the need to focus resources on the small group of patients who are likely to develop WS later with hospitalization requirement, to prevent severe disease progression. Lastly, innovation will be needed to reduce the cost and complexity of the current methods used to detect multiple RNA transcripts and protein simultaneously with a blood test based application, particularly for application in a developing countries.

## Conclusion

In summary, this is the first study, to our knowledge, that has shown adult dengue patients who later developed WS with hospitalization requirement have different pathophysiological features at Day 1–3 p.f. compared to adult dengue patients who did not develop WS and had no hospitalization requirement. Potential prognostic biomarkers models were developed from highly associated laboratory and molecular features, for triage at early infection, of adult dengue patients who are likely to develop WS later with hospitalization requirement. With future independent larger cohort for validation, these optimal models may be applied to complement the WHO 2009 dengue classifications. These biomarkers models would be best integrated with viral detection assays as a potential point-of care tool for both dengue diagnosis and disease prognosis, to guide clinical triage and treatment simultaneously and could be particularly useful if antivirals become available to treat dengue infection.

## Supporting Information

S1 Fig Laboratory characteristics of patients with severe dengue among the hospitalized dengue patients with warning signs (WS + Hosp. Group). (DOCX)

S2 Fig Laboratory characteristics of patients with severe dengue compared to non-hospitalized dengue patients with no warning signs (Non-WS + Non-Hosp. Group). (DOCX)

S1 Table Differential genes between hospitalized patients with warning signs (WS + Hosp.), including patients with severe dengue (SD) and non-hospitalized patients without warning signs (Non-WS + Non-Hosp.) at less than 72hr post fever. (DOCX)

S2 Table Targeted proteomic expression between Non-WS + Non-Hosp. group and WS + Hosp. group. (DOCX)

S3 Table Targeted proteomic expression between Non-WS + Non-Hosp. group and severe dengue patients among the WS + Hosp. group. (DOCX)
