# Supplementary material for: Discovery and Validation of Prognostic Biomarker Models to Guide Triage among Adult Dengue Patients at Early Infection
Source: PLoS One. 2016 Jun 10;11(6):e0155993. doi: 10.1371/journal.pone.0155993 (PMC4902184; doi:10.1371/journal.pone.0155993)
Supplement: S1 Fig — (DOCX) [file pone.0155993.s001.docx]

*
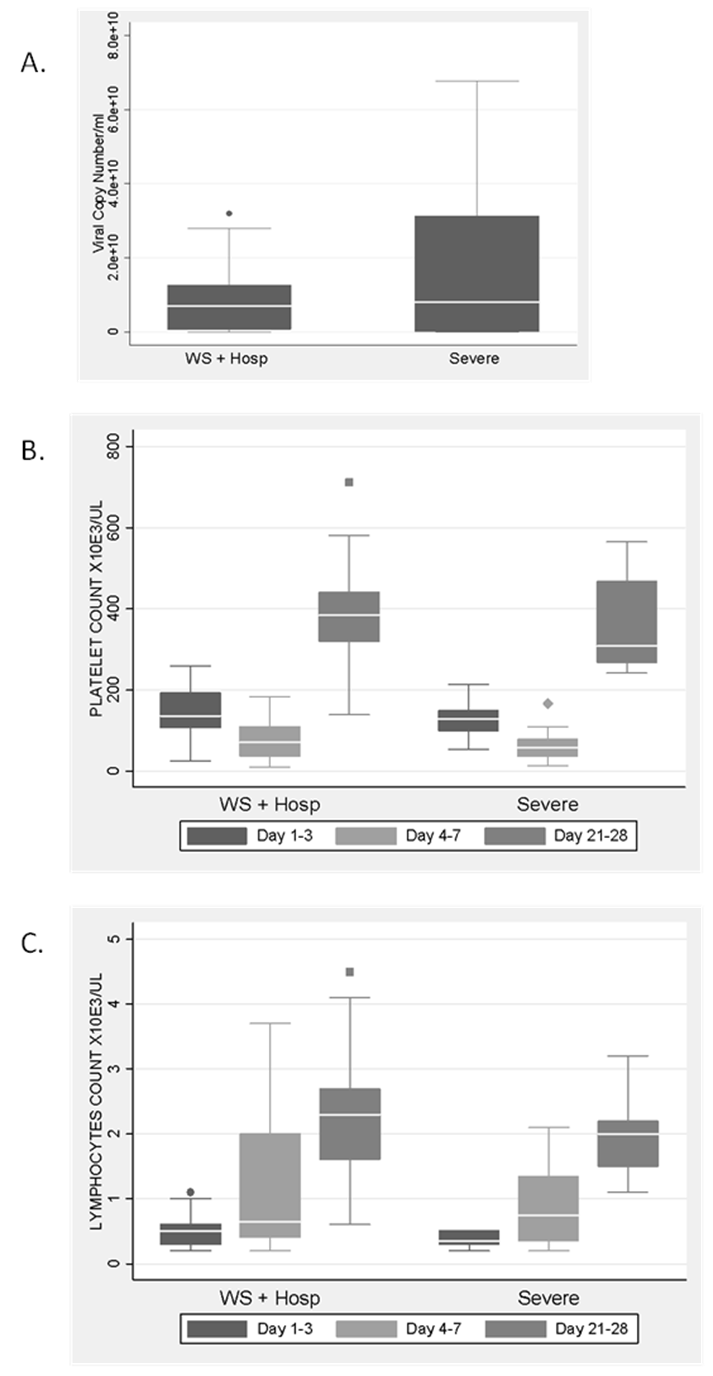
*

**S1 Figure. Laboratory characteristics of patients with severe dengue among the hospitalized dengue patients with warning signs (WS + Hosp. Group).**

P-value (P) is shown only for statistically significant comparisons on Day 1-3 and Day 4-7.
