# Supplementary material for: Discovery and Validation of Prognostic Biomarker Models to Guide Triage among Adult Dengue Patients at Early Infection
Source: PLoS One. 2016 Jun 10;11(6):e0155993. doi: 10.1371/journal.pone.0155993 (PMC4902184; doi:10.1371/journal.pone.0155993)
Supplement: S2 Fig — (DOCX) [file pone.0155993.s002.docx]

**
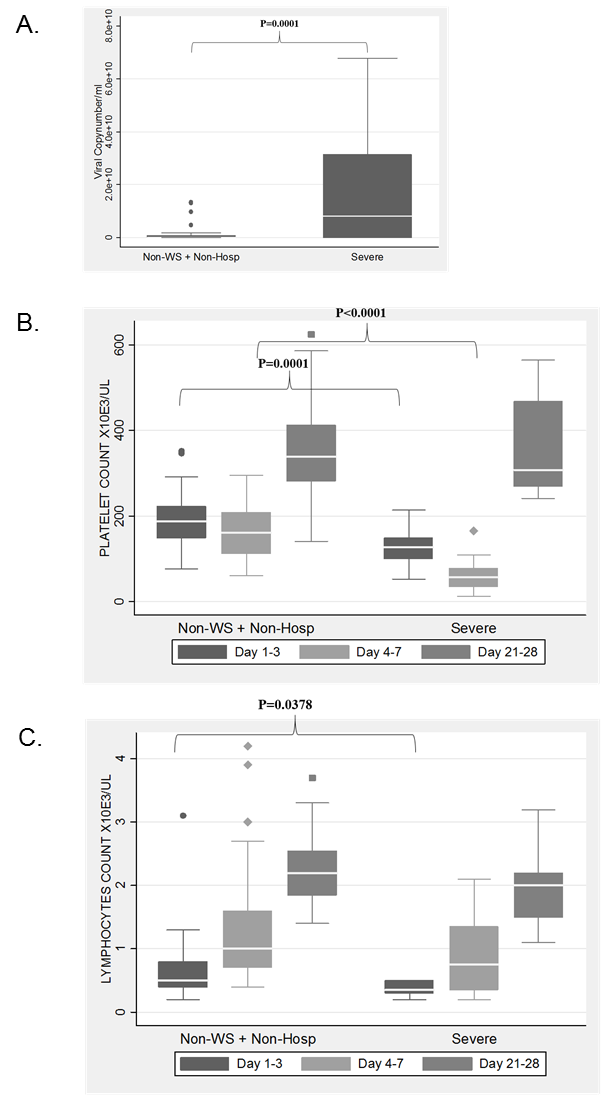
**

**S2 Figure. Laboratory characteristics of patients with severe dengue compared to non-hospitalized dengue patients with no warning signs (Non-WS + Non-Hosp. Group).** P-value (P) is shown only for statistically significant comparisons on Day 1-3 and Day 4-7.
