# Supplementary material for: Discovery and Validation of Prognostic Biomarker Models to Guide Triage among Adult Dengue Patients at Early Infection
Source: PLoS One. 2016 Jun 10;11(6):e0155993. doi: 10.1371/journal.pone.0155993 (PMC4902184; doi:10.1371/journal.pone.0155993)
Supplement: S1 Table — (DOCX) [file pone.0155993.s003.docx]

**S1 Table. Differential genes between hospitalized patients with warning signs (WS + Hosp.), including patients with severe dengue (SD) and non-hospitalized patients without warning signs (Non-WS + Non-Hosp.) at less than 72hr post fever.**

|  | **WS + Hosp. VS Non-WS + Non-Hosp.** | **SD VS Non-WS + Non-Hosp.** |  |  |
| --- | --- | --- | --- | --- |
| **Symbol** | **≥1.5 Fold change** | **≥2 Fold change** | **Acute vs convalecent^a^*** | **Dengue vs non dengue^b^** |
| IL-8 | - | 3.2 | N.S. | N.S. |
| PKD2L1 | 2.9 | 2.4 | Up reg. | Up reg. |
| CCL8 | 2.7 | 2.5 | Up reg. | Up reg. |
| HESX1 | 2.6 | - | Up reg. | Up reg. |
| BRDG1 | 2.5 | - | Up reg. | Up reg. |
| OLFM4 | 2.4 | - | N.S. | N.S. |
| CCL2 | 2.3 | 2.3 | Up reg. | Up reg. |
| SERPINE2 | - | 2.3 | N.S. | N.S. |
| CD69 | 2.2 | - | N.S. | N.S. |
| C15orf48 | 2.0 | 2.4 | N.S. | N.S. |
| ARL5B | 2.0 | - | N.S. | N.S. |
| RIN2 | 1.8 | - | Up reg. | Up reg. |
| HIST1H4E | 1.8 | - | N.S. | N.S. |
| NCOA7 | 1.6 | - | Up reg. | Up reg. |
| LOC440093 | 1.6 | - | N.S. | N.S. |
| KCTD14 | 1.6 | - | Up reg. | Up reg. |
| CCL3 | 1.6 | 2.2 | Up reg. | Up reg. |
| RGL1 | 1.6 | - | Up reg. | Up reg. |
| NFIL3 | 1.5 | - | Up reg. | N.S. |
| ESPN | -1.5 | - | Down reg. | N.S. |
| VPS13C | -1.6 | - | N.S. | N.S. |
| CYP27A1 | -1.6 | - | Down reg. | Down reg. |
| CDKN1C | -1.6 | - | Up reg. | Up reg. |
| SH3KBP1 | -1.8 | - | N.S. | N.S. |
| FECH | - | -2.2 | N.S. | N.S. |
| PI3 | -2.5 | - | Down reg. | Down reg. |

**^a^**Data from 32 patients generated by comparing samples from day 1-3 after fever onset with convalescent samples from day 21-28 after fever onset [[5](#_ENREF_5)].

^b^Data generated by comparing samples from dengue positive patients sampled day 1-3 after fever onset with samples from dengue negative febrile patients sampled day 1-3 after fever onset [[5](#_ENREF_5)].

Reg.- regulated

N.S.- not significant
