# Supplementary material for: Discovery and Validation of Prognostic Biomarker Models to Guide Triage among Adult Dengue Patients at Early Infection
Source: PLoS One. 2016 Jun 10;11(6):e0155993. doi: 10.1371/journal.pone.0155993 (PMC4902184; doi:10.1371/journal.pone.0155993)
Supplement: S2 Table — (DOCX) [file pone.0155993.s004.docx]

**S2 Table. Targeted proteomic expression between Non-WS + Non-Hosp. group and WS + Hosp. group.**

|  |  | **DAY 1- 3** | | | | | | |  |
| --- | --- | --- | --- | --- | --- | --- | --- | --- | --- |
| **Proteins** |  | **Non-WS + Non-Hosp** | | | **WS + Hosp** | | |  |  |
|  |  | **N** | **Mean** | **SD** | **N** | **Mean** | **SD** | **p-value** |  |
| **Chemokines** |  |  |  |  |  |  |  |  |  |
| **MCP-1 (CCL2)** |  | 45 | 351.29 | 382.65 | 47 | 537.16 | 701.5 | 0.117 |  |
| **MCP-2 (CCL8)** |  | 45 | 693.78 | 354.13 | 47 | 896.23 | 821.28 | 0.127 |  |
| **MIP1a (CCL3)** |  | 45 | 6.63 | 8.74 | 47 | 10.147 | 14.749 | 0.1661 |  |
| **MIP1b (CCL4)** |  | 45 | 252.48 | 151.46 | 47 | 325.81 | 225.47 | 0.0697 |  |
| **RANTES (CCL5)** |  | 26 | 16877 | 9103.6 | 27 | 11593 | 6900.1 | **0.0207** |  |
| **IP-10 (CXCL10)** |  | 45 | 10167 | 19784 | 46 | 23010 | 18217 | **0.0001** |  |
| **Interleukins** |  |  |  |  |  |  |  |  |  |
| **IL-1b** |  | 45 | 1.49 | 2.9855 | 39 | 1.6972 | 1.0811 | 0.6662 |  |
| **IL-1ra (IL1RN)** |  | 45 | 1362.4 | 1524.6 | 47 | 3707.6 | 5757.8 | **0.0094** |  |
| **IL-2** |  | 45 | 6.32 | 17.604 | 47 | 7.7313 | 12.831 | 0.6617 |  |
| **IL-4** |  | 45 | 0.68 | 2.2777 | 47 | 0.6202 | 0.3982 | 0.8731 |  |
| **IL-8** |  | 45 | 18.55 | 9.3792 | 47 | 26.944 | 34.811 | 0.1171 |  |
| **IL-10** |  | 45 | 44.04 | 85.912 | 47 | 53.634 | 94.401 | 0.6118 |  |
| **IL-12** |  | 45 | 9.48 | 11.499 | 47 | 7.4145 | 5.9815 | 0.286 |  |
| **IL-18** |  | 35 | 228.41 | 113.43 | 43 | 281.46 | 157.41 | 0.0988 |  |
| **Interferons** |  |  |  |  |  |  |  |  |  |
| **IFN-a2** |  | 45 | 112.91 | 74.972 | 47 | 121.91 | 61.267 | 0.529 |  |
| **IFN-g** |  | 45 | 60.488 | 95.614 | 47 | 76.695 | 89.56 | 0.4034 |  |
| **Tumor Necrosis Factor** |  |  |  |  |  |  |  |  |  |
| **TNF-a (TNF)** |  | 45 | 24.829 | 18.477 | 47 | 32.323 | 26.032 | 0.114 |  |
| **TRAIL(TNFSF10)** |  | 45 | 994.08 | 525.45 | 47 | 1060.3 | 538.45 | 0.5525 |  |
| **Others** |  |  |  |  |  |  |  |  |  |
| **Fibrinogen (FGA)** |  | 35 | 4820.7 | 3476.7 | 35 | 7672.3 | 7304.2 | **0.0423** |  |
| **ICAM-1** |  | 26 | 231917 | 64163 | 27 | 260042 | 97853 | 0.2207 |  |
| **VCAM-1** |  | 26 | 409475 | 227158 | 27 | 528265 | 252640 | 0.0781 |  |
| **uPAR (PLAUR)** |  | 35 | 5870.5 | 1990.3 | 35 | 7786.4 | 3298.2 | **0.0047** |  |
