# Supplementary material for: Discovery and Validation of Prognostic Biomarker Models to Guide Triage among Adult Dengue Patients at Early Infection
Source: PLoS One. 2016 Jun 10;11(6):e0155993. doi: 10.1371/journal.pone.0155993 (PMC4902184; doi:10.1371/journal.pone.0155993)
Supplement: S3 Table — (DOCX) [file pone.0155993.s005.docx]

**S3 Table. Targeted proteomic expression between Non-WS + Non-Hosp. group** **and severe dengue patients among the WS + Hosp. group.**

|  |  | **DAY 1- 3** | | | | | | |  |
| --- | --- | --- | --- | --- | --- | --- | --- | --- | --- |
| **Proteins** |  | **Non-WS + Non-Hosp** | | | **Severe Dengue** | | | |  |
|  |  | **N** | **Mean** | **SD** | **N** | **Mean** | **SD** | **p-value** |  |
| **Chemokines** |  |  |  |  |  |  |  |  |  |
| **MCP-1 (CCL2)** |  | 45 | 351.2893 | 382.6473 | 14 | 822.98 | 1119.20 | 0.159 |  |
| **MCP-2 (CCL8)** |  | 45 | 693.78 | 354.13 | 14 | 1015.52 | 761.18 | 0.162 |  |
| **MIP1a (CCL3)** |  | 45 | 6.629556 | 8.74475 | 14 | 12.23 | 16.49 | 0.258 |  |
| **MIP1b (CCL4)** |  | 45 | 252.4771 | 151.4567 | 14 | 433.63 | 289.68 | **0.048** |  |
| **RANTES (CCL5)** |  | 26 | 16876.54 | 9103.565 | 7 | 11405.57 | 7966.89 | 0.147 |  |
| **IP-10 (CXCL10)** |  | 45 | 10166.67 | 10784.03 | 14 | 32301.95 | 22130.61 | **0.004** |  |
| **Interleukins** |  |  |  |  |  |  |  |  |  |
| **IL-1b** |  | 45 | 1.49 | 2.99 | 10 | 1.92 | 1.44 | 0.507 |  |
| **IL-1ra (IL1RN)** |  | 45 | 1362.43 | 1524.61 | 14 | 5787.56 | 8647.18 | 0.091 |  |
| **IL-2** |  | 45 | 6.32 | 17.60 | 14 | 6.17 | 8.56 | 0.968 |  |
| **IL-4** |  | 45 | 0.68 | 2.28 | 14 | 0.74 | 0.55 | 0.858 |  |
| **IL-8** |  | 45 | 18.55 | 9.38 | 14 | 41.55 | 63.10 | 0.116 |  |
| **IL-10** |  | 45 | 44.04 | 85.91 | 14 | 38.92 | 30.23 | 0.739 |  |
| **IL-12** |  | 45 | 9.48 | 11.50 | 14 | 7.48 | 3.11 | 0.301 |  |
| **IL-18** |  | 35 | 228.41 | 113.43 | 14 | 338.87 | 227.58 | 0.116 |  |
| **Interferons** |  |  |  |  |  |  |  |  |  |
| **IFN-a2** |  | 45 | 112.9089 | 74.9718 | 14 | 133.62 | 57.89 | 0.300 |  |
| **IFN-g** |  | 45 | 60.48844 | 95.6135 | 14 | 71.24 | 43.59 | 0.568 |  |
| **Tumor Necrosis Factor** |  |  |  |  |  |  |  |  |  |
| **TNF-a (TNF)** |  | 45 | 24.82933 | 18.47697 | 14 | 31.05 | 16.86 | 0.265 |  |
| **TRAIL(TNFSF10)** |  | 45 | 994.0789 | 525.9553 | 14 | 1233.53 | 587.51 | 0.202 |  |
| **Others** |  |  |  |  |  |  |  |  |  |
| **Fibrinogen (FGA)** |  | 35 | 4820.73 | 3476.66 | 10 | 10825.44 | 10865.57 | 0.117 |  |
| **ICAM-1** |  | 26 | 231916.5 | 64162.6 | 7 | 257704.26 | 104522.33 | 0.553 |  |
| **VCAM-1** |  | 26 | 409475 | 227158.3 | 7 | 510908.98 | 264411.17 | 0.379 |  |
| **uPAR (PLAUR)** |  | 35 | 5870.54 | 1990.26 | 10 | 8522.53 | 3022.12 | **0.023** |  |
